# Improved lung preservation relates to an increase in tubular myelin-associated surfactant protein A

**DOI:** 10.1186/1465-9921-6-60

**Published:** 2005-06-21

**Authors:** Heinz Fehrenbach, Sebastian Tews, Antonia Fehrenbach, Matthias Ochs, Thorsten Wittwer, Thorsten Wahlers, Joachim Richter

**Affiliations:** 1 Division of Electron Microscopy, Centre of Anatomy, University of Göttingen, Kreuzbergring 36, D-37075 Göttingen, Germany; 2 Clinical Research Group "Chronic Airway Diseases", Department of Internal Medicine (Respiratory Medicine), Philipps-University, Baldingerstrasse, D-35043 Marburg, Germany; 3 Department of Cardiothoracic and Vascular Surgery, Friedrich Schiller University Jena, Bachstrasse 18, D-07740 Jena, Germany; 4 Institute of Anatomy, University of Bern, Baltzerstrasse 2, CH-3000 Bern 9, Switzerland

## Abstract

**Background:**

Declining levels of surfactant protein A (SP-A) after lung transplantation are suggested to indicate progression of ischemia/reperfusion (IR) injury. We hypothesized that the previously described preservation-dependent improvement of alveolar surfactant integrity after IR was associated with alterations in intraalveolar SP-A levels.

**Methods:**

Using immuno electron microscopy and design-based stereology, amount and distribution of SP-A, and of intracellular surfactant phospholipids (lamellar bodies) as well as infiltration by polymorphonuclear leukocytes (PMNs) and alveolar macrophages were evaluated in rat lungs after IR and preservation with EuroCollins or Celsior.

**Results:**

After IR, labelling of tubular myelin for intraalveolar SP-A was significantly increased. In lungs preserved with EuroCollins, the total amount of intracellular surfactant phospholipid was reduced, and infiltration by PMNs and alveolar macrophages was significantly increased. With Celsior no changes in infiltration or intracellular surfactant phospholipid amount occurred. Here, an increase in the number of lamellar bodies per cell was associated with a shift towards smaller lamellar bodies. This accounts for preservation-dependent changes in the balance between surfactant phospholipid secretion and synthesis as well as in inflammatory cell infiltration.

**Conclusion:**

We suggest that enhanced release of surfactant phospholipids and SP-A represents an early protective response that compensates in part for the inactivation of intraalveolar surfactant in the early phase of IR injury. This beneficial effect can be supported by adequate lung preservation, as e.g. with Celsior, maintaining surfactant integrity and reducing inflammation, either directly (via antioxidants) or indirectly (via improved surfactant integrity).

## Background

Surfactant protein A (SP-A) is the major surfactant-associated protein, and is of central importance to the structure, metabolism, and function of pulmonary surfactant (as reviewed by [1-3]). It is also important for the regulation of inflammatory processes and for innate host defence of the lung (as reviewed by [4]).

Reduced intraalveolar levels of SP-A were found to be associated with several pulmonary diseases [5,6]. In the pathological situation, SP-A is therefore suggested to be an important regulator of surfactant function. In lung transplant recipients, impairment of pulmonary surfactant activity was associated with an increased ratio of small-to-large surfactant aggregates and a reduced content of SP-A [7,8]. Using an extracorporeal model of ischemia/reperfusion (IR) injury in the rat lung, we showed preservation-dependent alterations in the ratio between inactive (unilamellar vesicles) and active (tubular myelin) surfactant components [9]. Based on these studies, we hypothesize preservation-dependent effects on the amount and distribution of intraalveolar SP-A. We further propose that the preservation-dependent differences in the amount of surface active surfactant in the alveoli are associated with alterations of the intracellular surfactant pool.

An established extracorporeal rat lung model was used to study the cumulative effects induced by the whole sequence of transplantation-related events, which includes flush perfusion, cold ischemic storage, and subsequent reperfusion of the lung, rather than looking at the relative contribution of the individual events. The quality of preservation by the solutions, EuroCollins and Celsior, was compared using established stereological methods [10,11]. These design-based techniques allow for a quantitative structural analysis in the organ by light and electron microscopy. The methods are unbiased, efficient, and representative for the whole lung (for review see [12]).

## Methods

### Animals

Twenty-four male Sprague-Dawley rats (Crl:CD; Charles River, Sulzfeld, Germany) received pentobarbital intraperitoneally (Nembutal 1 mg/kg body weight), were intubated by tracheostomy, and heparinized via the vena cava inferior. Animal experiments were performed according to the Helsinki convention for the use and care of animals. The experiments have been approved by the regional government.

### Study design

The study was particularly designed to investigate if EuroCollins and Celsior solution were able to adequately preserve the levels of surfactant protein A (SP-A) and of the intracellular surfactant phospholipid stores. In order to consider a preservation solution as adequate, it should be effective throughout the periods of ischemia and reperfusion in maintaining levels, which are characteristic for a native lung. Therefore, two separate sets of experiments were performed: 1) preparation for SP-A analysis by immuno electron microscopy (n = 3 per group) and 2) preparation for surfactant phospholipid analysis by conventional transmission electron microscopy (n = 5 per group). Each experimental set comprised three groups: 1) controls: no intervention (native lungs), 2) EuroCollins: flush perfusion with Euro Collins solution containing 40 mMol potassium (EC40) supplemented with 6 μg/100 ml prostacyclin (Epoprostenol; Flolan, Wellcome, Beckenham, UK), and 3) Celsior: flush perfusion with Celsior (IMTIX, Pasteur Merieux, France); both 2) and 3) with 120 minutes of ischemia (at 10°C), and 50 minutes of reperfusion.

### Extracorporeal model of ischemia/reperfusion injury

Operation and excision of the heart-lung-block was performed as described recently [13]. Lungs were flushed via the pulmonary artery at a hydrostatic pressure of 20 cm H_2_O with preservation solution (for composition, see Table 1). Ischemic storage (120 min) was followed by a 50-min reperfusion via the pulmonary artery with Krebs-Henseleit-buffer (8.0 ml/min at 37°C) containing bovine red blood cells (hematocrit of 38 to 40%) using a quattro head roller pump (Mod-Reglo-Digital, Ismatec, Zürich, Switzerland).

**Table 1:** Composition of Preservation solutions

**Components**	**EuroCollins [mmol/l]**	**Celsior^® ^[mmol/l]**
Na^+^	85	100
K^+^	40	15
Mg^2+^	-	13
Ca^2+^	-	0.26
Cl^-^	15	41.5
PO_4_^2-^	57.5	-
HCO^3-^	10	-
Histidine	-	30
Mannitol	-	60
Glucose	3.5 [%]	-
Glutamate	-	20
Lactobionate	-	80
Glutathione	-	3

osmolarity	370	360

### Lung function measurements

Perfusate oxygenation (ΔPO_2_), peak inspiratory pressure (PIP) as well as pulmonary arterial pressure (PAP) were measured at the end of the reperfusion period of 50 minutes as described earlier [9].

### Fixation, tissue sampling and processing

Fixation by vascular perfusion and tissue sampling as well as tissue processing for standard and immuno electron microscopy have been described previously [9,14,15]. Lung volume was determined and isotropic uniform random samples (IUR) of lung tissue were taken and processed according to standard methods [14]. The tissue samples were embedded either in glycolmethacrylate resin (Technovit 7100, Heraeus, Kulzer, Germany) for light microscopy, or in Araldite for electron microscopy.

For immuno electron microscopy, lungs were fixed with 4% paraformaldehyde/ 0.1% glutardialdehyde in 0.2 M Hepes buffer. After collection of IUR tissue samples (see above), 2 mm^3 ^tissue blocks were infiltrated in 2.3 M sucrose in PBS for at least 1 hour and frozen in liquid nitrogen, then freeze-substituted (Reichert AFS; Leica, Vienna, Austria) in 0.5% uranyl acetate in methanol at -90°C for at least 36 hours and embedded in Lowicryl HM20 (Polysciences, Eppelheim, Germany) at -45°C (for details see [14]).

### Immunolabelling

Ultrathin sections (70 nm thickness) were labelled with affinity purified polyclonal primary antibody against SP-A (dilution 1:40 for labelling of type II pneumocytes and 1:150 for labelling of tubular myelin; kind gift from Dr. S. Hawgood, San Francisco) and gold-coupled secondary antibody (dilution 1:20; British Biocell; Cardiff, UK) with a gold particle diameter of 10 nm for detection. Control experiments were performed by omission of the primary antibody. Immunolabelling was examined using an EM 900 (LEO, Oberkochen, Germany) at a magnification of × 20,000.

### Stereological analysis of SP-A labelling

The numbers of gold labelling on tubular myelin as well as on nucleus, mitochondria, lamellar bodies and the remaining cytoplasm (including vesicles) of type II pneumocytes were counted and related to the volume fraction of the cellular compartments and to the length of tubular myelin phospholipid layers as described by Griffiths [16]. The relative labelling index (RLI), was determined to test for preferential labelling of different cell compartments according to a recently described method, which allows for clearer distinction between specific labelling and unspecific background staining [15,17]. A total of 172 profiles of alveolar epithelial type II cells were analyzed. The total number of gold particles counted over alveolar epithelial type II cell profiles was 10,530, thus the mean number of gold particles counted per cell profile was 61.

Using intersection counting, labelling density of SP-A over the tubular myelin lattices was determined as particle number referred to the length of the profile of the phospholipid layers forming the lattice according to the formula: N_gold _/ length = N_gold _/ I × d with number of intersections (I) and distance between the test lines (d). Using point counting, the labelling density of SP-A over type II cell profiles was determined according to the formula: N_gold _= N_gold _/ p × d^2 ^with number of points (p) and distance between the test lines (d). Due to the dependence of the effective resolution of gold labelling on the size of the underlying particles [16], we did not choose to separate the vesicles from the cytoplasmic compartment to avoid uncertainties and misinterpretations in the allocation of the gold particles.

### Stereological analysis of alveolar epithelial type II cell parameters and lamellar bodies

Number and volume of alveolar epithelial type II cells (AEC II) as well as number, size, and volume of lamellar bodies were quantified on a computer-assisted light microscope (Cast-Grid 2.0, Olympus, Denmark) using the physical disector, rotator, and point-sampled intercepts method as previously described in detail [11]. AEC II (93 ± 9^SEM ^per lung) were sampled by light microscopy on glycolmethacrylate sections using the single section disector [12].

**Figure 1:**
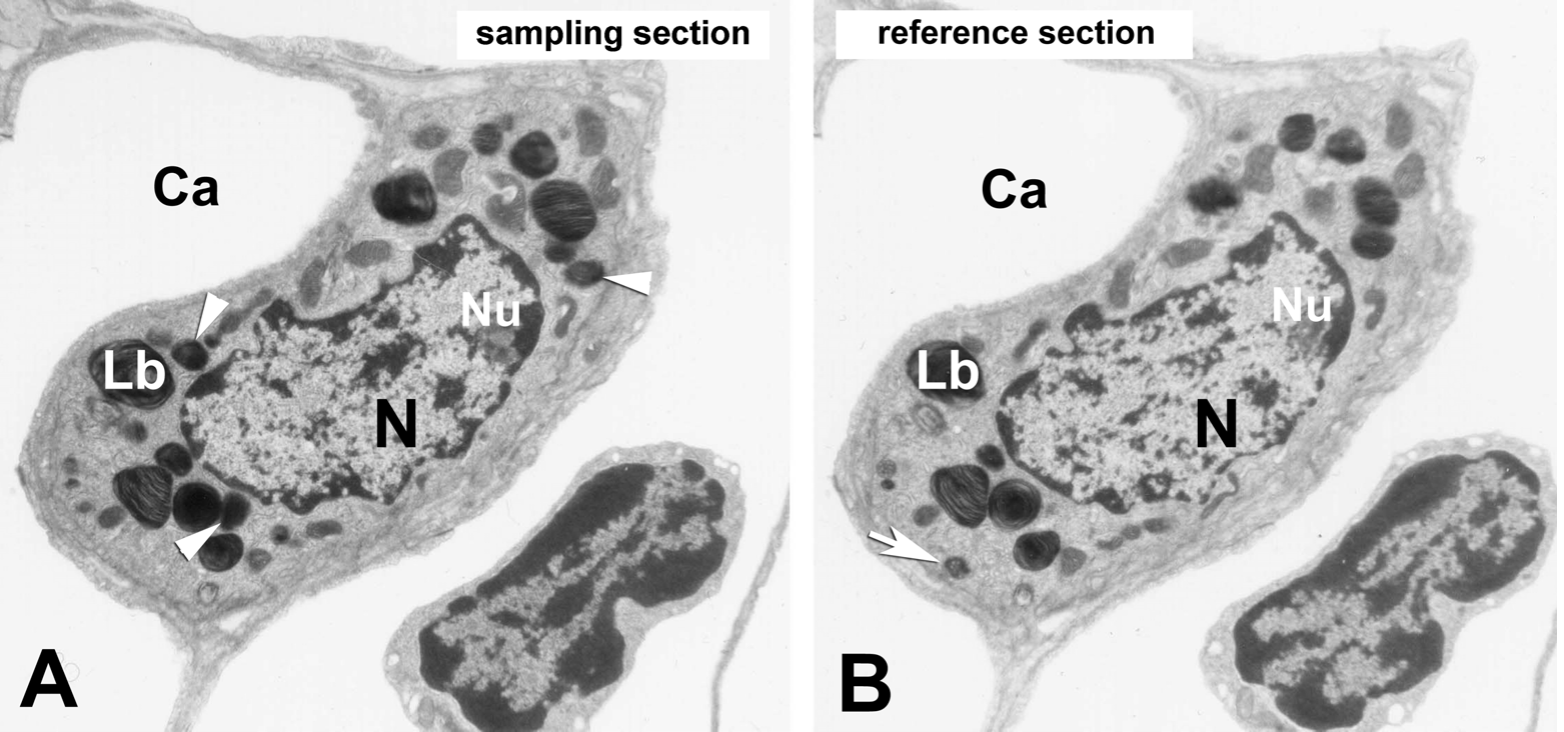
**Principle of Physical Disector**. Electron micrographs showing sets of two parallel sections (~100 nm thick) of an alveolar epithelial type II cell. Three lamellar bodies (arrowheads), which only occur in the sampling section, were counted as well as one lamellar body (arrow) seen in the reference section, because the principle of bidirectional counting was applied. Nucleus (N), nucleolus (Nu), lamellar body (Lb), capillary (Ca).

According to Ochs and co-workers [10], the physical disector was used for counting the number of lamellar bodies and of type II pneumocytes, which allows for quantification of the intracellular pool of surfactant phospholipids per cell and per unit lung volume. Disector counting of lamellar bodies was performed on sets of two parallel ultrathin sections with a known separation of approximately 100 nm (estimated by the Small fold method according to [18]), the reference and the look-up or sampling section (Fig. 1).

**Figure 2:**
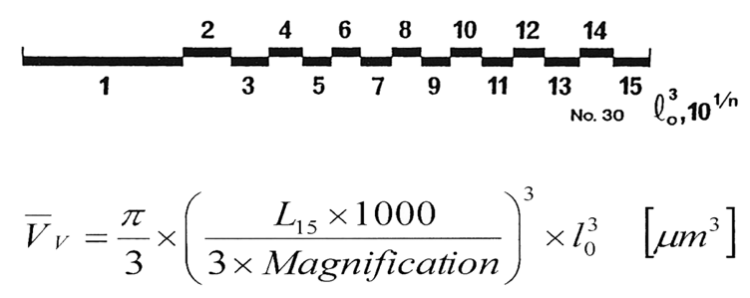
**Logarithmic Ruler**. Logarithmic ruler and formula for the determination of the volume weighted mean volume () of lamellar bodies according to Brændgaard and Gundersen [37].

The apical (secretory) fraction of the AEC II surface (S_S_) and the mean volume-weighted particle volume () of lamellar bodies was determined on electron micrographs (magnification ×7500) of AEC II, which had been sampled in a systematic uniform random manner, by means of the point-sampled intercept method [11] (Fig. 2). The number-weighed mean volume (V_NLb_) of lamellar bodies was calculated by dividing the total volume of lamellar bodies (V_Lb_ per cell by the total number (N_Lb_) of lamellar bodies per cell.

### Stereological analysis of polymorphonuclear leukocytes (PMN) and alveolar macrophages

The volume densities and total volume of PMNs and alveolar macrophages in lung parenchyma was evaluated by point counting according to standard methods [14] using computer-assisted light microscopy (Cast-Grid 2.0, Olympus, Denmark).

### Statistics

Differences between the experimental groups and the control group were tested for significance with parametric One Way ANOVA followed by post hoc multiple comparisons (Dunnett's method) provided that normality and equal variance given at p>0.1 were given. The differences in the size classes of lamellar bodies were tested by Mann-Whitney-U test. Otherwise, non-parametric Mann-Whitney rank sum test or Kruskal-Wallis One Way ANOVA on ranks was used. Mean values are given ± SEM unless otherwise indicated. Preferential or specific labelling for SP-A was tested by χ^2^-analysis [17]. Correlations between stereological and lung function parameters were tested by multivariate analysis using forward stepwise regression to identify those stereological parameters that were predictors of ΔPO_2_, PIP, and PAP, respectively. All statistical analyses and graphic presentations were performed using the SigmaStat2.0 and SigmaPlot8.0 software programs (Jandel Scientific, Erkrath, Germany). *p *values < 0.05 were considered to be significant unless otherwise indicated.

## Results

### Surfactant protein A

**Figure 3:**
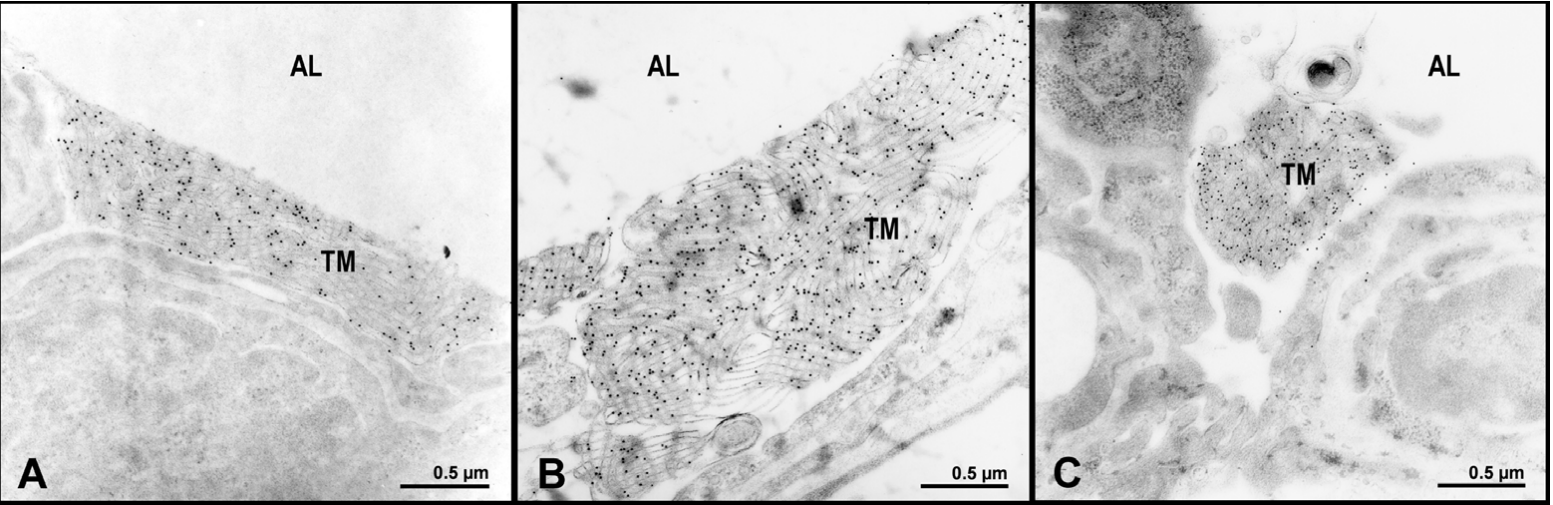
**Intact tubular myelin immunolabelled for SP-A**. Immunolabelling for SP-A on ultrastructurally intact tubular myelin (TM) lattices A) in the control, B) after ischemia and reperfusion following preservation with either Celsior or C) EuroCollins. Alveolar lumen (AL), epithelium (EPI).

**Figure 4:**
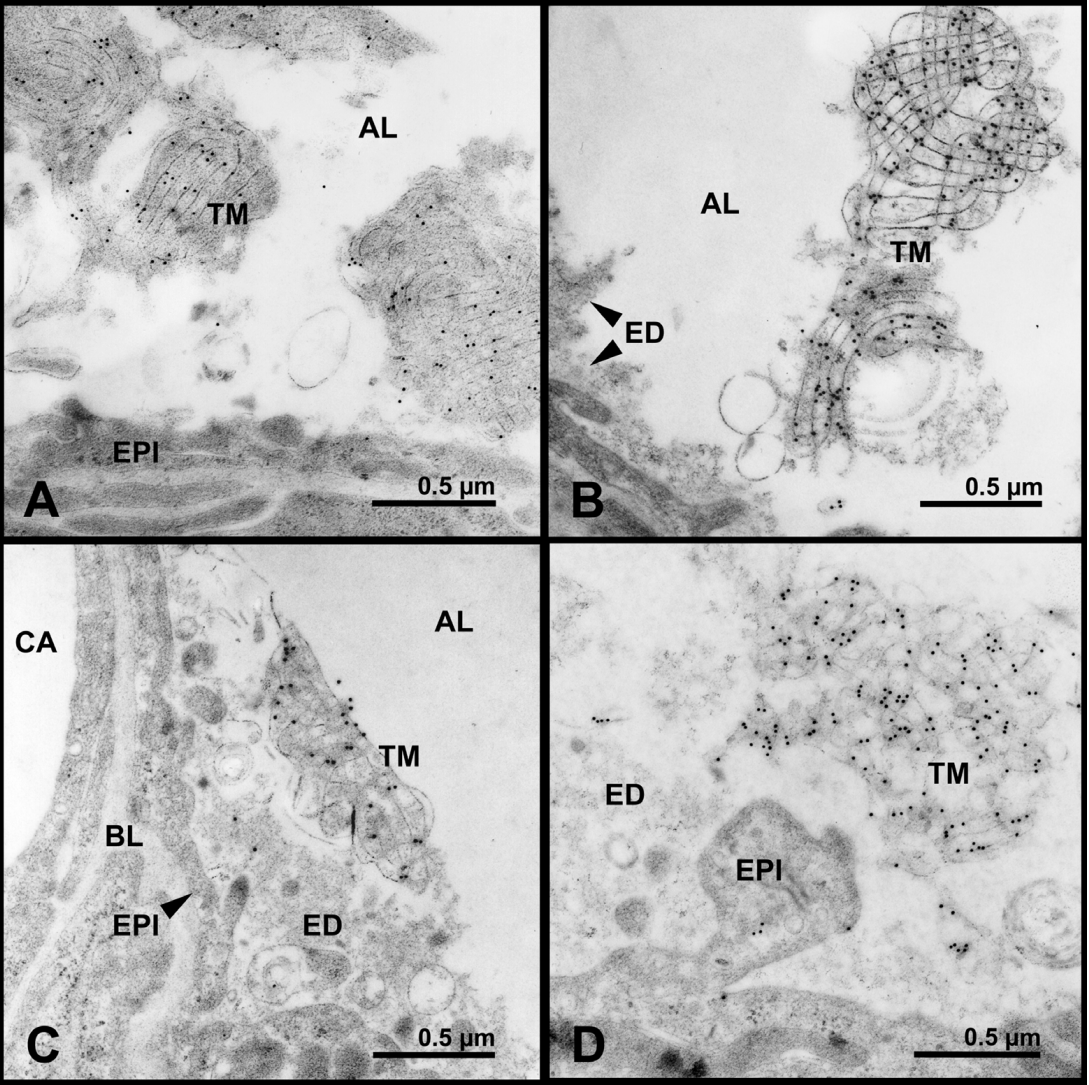
**Altered tubular myelin immunolabelled for SP-A**. Immunolabelling for SP-A on altered tubular myelin (TM) lattices: A) tubular myelin is dislocated from the alveolar wall in a control lung and B) after ischemia and reperfusion following preservation with Celsior; C) and D) side dimensions of the tubular myelin lattices is enlarged after ischemia and reperfusion following preservation with either Celsior (C) or EuroCollins (D). Alveolar lumen (AL), basal lamina (BL), capillary (CA), edema (ED), epithelium (EPI).

**Figure 5:**
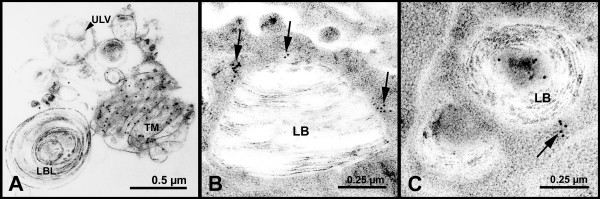
**Surfactant subtypes immunolabelled for SP-A**. Specific labelling for SP-A did not occur on A) unclustered lamellar body-like surfactant forms (LBL) nor unilamellar vesicles (ULV); B) cytoplasm/multivesicular bodies (arrows) displayed specific labelling for SP-A (RLI ≌ 1.57) whereas C) the weak labelling of intracellular lamellar bodies (LB) was non-specific (RLI ≌ 0.34). Tubular myelin (TM).

Labelling for SP-A was strongest over the lattice structures of tubular myelin figures in all study groups and was significantly increased in lungs after ischemia and reperfusion (IR) (Table 2; Fig. 3). Characteristic alterations of the tubular myelin ultrastructure, e.g. enlargement of the side dimensions of the tubular myelin lattices, termed as mesh width, appearance of unilamellar vesicles among disintegrating lattices, and dislocation of tubular myelin from the alveolar wall could either be accompanied by weak or by strong labelling for SP-A without any preferential association (Fig. 4). Densely clustered intraalveolar lamellar body-like forms showed SP-A labelling over peripheral lamellae, whereas unclustered forms as well as unilamellar vesicles did not display any labelling for SP-A (Fig. 5A).

**Table 2:** Characteristics of Tubular Myelin Ultrastructure and Labelling Density of Surfactant Protein A (SP-A)

**Parameter**	**Control**	**EuroCollins**	**Celsior**
Mesh width of tubular myelin [nm]	30.6 ± 3.3	43.8 ± 1.3*	32.7 ± 4.0
Number of gold particles (SP-A) on tubular myelin^1 ^[μm^-1^]	11.1 ± 1.5	31.9 ± 3.5*	35.9 ± 0.2*
Number of gold particles (SP-A) on AEC II cytoplasm^2 ^[μm^-2^]	4.2 ± 0.4	3.1 ± 0.3^#^	2.9 ± 0.2^#^

means ± SEM of n = 3 per group; *p < 0.05 and ^#^0.05 < p < 0.1 versus control

^1 ^gold particle counts are referred to the length of the phospholipid layer cross sections composing the tubular myelin lattices

^2 ^gold particle counts are referred to the sectioned area of AEC II

Within alveolar epithelial type II cells, SP-A was localized mainly in small vesicles and multivesicular bodies close to the lamellar bodies (Fig. 5B, C). Labelling of lamellar bodies was rare and was usually associated with an electron dense area (Fig. 5C). Estimation of the relative labelling index (RLI) revealed a highly significant (p < 0.001) non-random labelling for the cytoplasm in all three groups (see Additional File 1). Cytoplasmic labelling for SP-A was below control value after IR, but differences between the groups achieved a level of significance of 0.05 < p < 0.1 only (Table 2).

### Surfactant phospholipid structures

In lungs that had been preserved with EuroCollins, the side dimensions of the tubular myelin lattices (mesh width) were significantly increased compared to control lungs. After preservation with Celsior, changes in the lattice microstructure were quite variable and, in contrast to previous data [9], the tubular myelin mesh width did not show any significant alteration compared to the other groups (Table 2; Fig. 4B).

**Figure 6:**
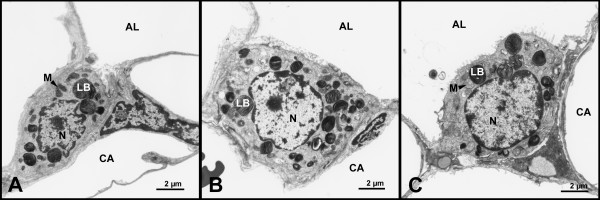
**Ultrastructural appearance of alveolar epithelial type II cells**. Alveolar epithelial type II cells differ in cell size as well as size and amount of lamellar bodies (LB) in A) the control and B) after ischemia and reperfusion following preservation with either Celsior or C) EuroCollins. Nuclei (N) and mitochondria (M) display edematous swelling in the treatment groups. Alveolar lumen (AL), capillary (CA).

**Figure 7:**
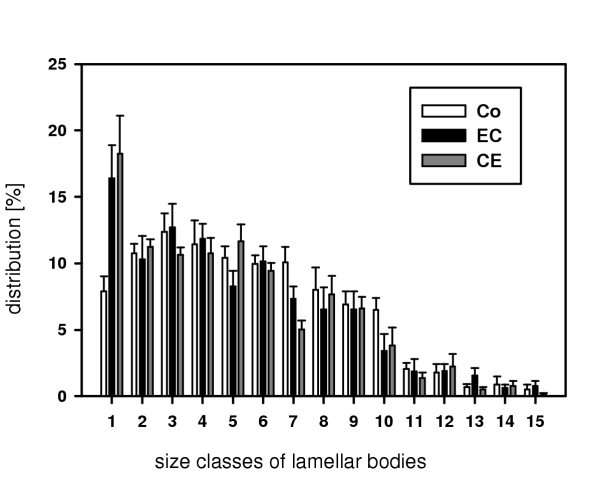
**Histogram of lamellar body size classes**. Distribution of lamellar body size classes (increasing from 1 to 15) after ischemia and reperfusion (IR) following preservation with either Celsior or EuroCollins compared to control lungs as determined by the point sampled intercepts method. After IR, lamellar bodies of size class1 (smallest size) differ significantly from controls (Celsior: p = 0.032; EuroCollins: p = 0.028). Bars represent means ± SD.

The total volume of lamellar bodies (V_Lb_) per lung was significantly decreased in lungs preserved with EuroCollins solution as compared with the control group (Table 3). This was accompanied by a decrease in the volume of lamellar bodies (V_Lb_) per type II cell, which, however, was not statistically significant (Table 3; Fig. 6C). There was no difference in the amount of intracellular surfactant (per lung as well as per cell) between the Celsior and the control groups (Table 3; Fig. 6A, B). In lungs preserved with Celsior, there was a significant reduction in the number-weighted mean volume () of lamellar bodies in comparison to the control group (Table 3). This was accompanied by a significant increase in the fraction of small section profiles of lamellar bodies after IR compared to controls (Fig. 7).

**Table 3:** Characteristics of lamellar bodies (Lb)

**Parameter**	**Control**	**EuroCollins**	**Celsior**
Number (N_Lb_) / AEC II	92.9 ± 5.4	97.9 ± 13.2	121.2 ± 10.0
Volume (V_Lb_) / AEC II [μm^3^]	58.2 ± 2.4	50.1 ± 3.2	57.4 ± 5.1
Total Volume (V_Lb_) per lung [10^9 ^μm^3^]	9.0 ± 0.9	5.4 ± 0.6*	6.3 ± 0.8
Mean Volume_number-weighted _() [μm^3^]	0.63 ± 0.06	0.55 ± 0.18	0.48 ± 0.10*

means ± SEM of n = 5 per group; *p < 0.05 versus control

### Alveolar epithelial type II cells

Type II cells as well as their subcellular compartments displayed significant oedematous swelling in lungs after IR as indicated by markedly increased volumes of the cells, cytoplasm, nuclei, and mitochondria compared to the controls (Table 4; Fig. 6). The surface fraction of the apical secretory surface of type II pneumocytes was unchanged after IR (Table 4).

**Table 4:** Characteristics of Type II Alveolar epithelial cells (AEC II)

**Parameter**	**Control**	**EuroCollins**	**Celsior**
**Apical surface fraction**			
(S_S_apical surface per total AEC II surface) [%]	47.8 ± 1.9	47.1 ± 1.4	47.3 ± 1.2
**Volumes [μm^3^]**			
Total Cell (V_AECII_)	322.5 ± 9.6	485.4 ± 33.7*	496.2 ± 37.4*
Cytoplasm (V_Cyt_) AECII	178.0 ± 5.1	279.6 ± 16.3*	279.2 ± 24.3*
Nucleus (V_Nu_) / AEC II	65.0 ± 3.9	117.5 ± 15.6*	125.0 ± 9.3*
Mitochondria (V_Mito_) / AEC II	21.4 ± 1.4	38.2 ± 4.6*	34.6 ± 2.2*

means ± SEM of n = 5 per group; *p < 0.05 versus control

### Polymorphonuclear leukocytes (PMN) and alveolar macrophages

Total volumes as well as volume densities of PMN (residing in the capillary bed) and alveolar macrophages (in the alveolar space) in the gas-exchange region were significantly increased (p < 0.05) after preservation with EuroCollins as compared with control lungs (Table 5). In lungs preserved with Celsior, PMN volume was similar to control lungs.

**Table 5:** Characteristics of polymorphonuclear leukocytes and alveolar macrophages in lung parenchyma

**Parameter**	**Control**	**EuroCollins**	**Celsior**
**Total Volume [mm_3_]**			
PMNs	1.7 ± 0.40	14.3 ± 10.0*	2.2 ± 0.6
Macrophages	< 0.01	6.3 ± 1.6*	1.2 ± 0.2
**Volume density [mm^3^/mm^3^]**			
PMNs	0.03 ± 0.01	0.36 ± 0.25*	0.05 ± 0.01
Macrophages	< 0.01	0.15 ± 0.03*	0.03 ± 0.01

means ± SEM of n = 5 per group; *p < 0.05 versus control

### Structure-function correlations

The quantitative-morphological parameters given in Tables 3, 4 and 5 were tested for potential correlation with the lung function parameters recorded at the end of the reperfusion period, i.e. immediately prior to fixation (Table 6). Multivariate regression analysis revealed that PIP can be predicted from a linear combination of the total volume of alveolar macrophages (r^2 ^= 0.514; p = 0.005) and the number of lamellar bodies (r^2 ^= 0.843; Δr^2 ^= 0.329; p = 0.006). ΔPO_2 _can be predicted from a linear combination of the total alveolar macrophage volume (r^2 ^= 0.536; p < 0.001) and total lamellar body volume (r^2 ^= 0.862; Δr^2 ^= 0.326; p = 0.005). There were no correlations between lung function parameters and PMNs or other AECII related parameters.

**Table 6:** Lung function characteristics after 50 min of reperfusion

**Parameter**	**EuroCollins**	**Celsior**
Perfusate ΔPO_2 _[mm Hg]	38.5 ± 8.2	126.0 ± 14.5**
Peak inspiratory pressure [cm H_2_O]	15.3 ± 2.2	11.2 ± 1.0
Pulmonary arterial pressure [cm H_2_O]	10.7 ± 1.2	9.4 ± 0.7

means ± SEM of n = 5 per group; ** p < 0.001 versus EuroCollins

## Discussion

We hypothesized that the previously described preservation-dependent improvement of alveolar surfactant integrity after ischemia and reperfusion (IR) [9] was associated with changes in the amount and distribution of SP-A as well as with alterations in the intracellular surfactant pool of alveolar epithelial type II cells. Using immuno electron microscopy, we showed that the labelling density of tubular myelin-associated SP-A was significantly enhanced after IR, and that the previously reported increase of the intraalveolar surfactant phospholipids [9] was paralleled by a trend to decreased intracellular SP-A levels. The total volume of intracellular surfactant phospholipids was significantly decreased in lungs perfused with EuroCollins, whereas lungs preserved with Celsior did not significantly differ from control lungs. The maintenance of intracellular surfactant in Celsior preserved lungs was achieved by an increase in the lamellar body number per alveolar epithelial type II cells despite a significant decrease in the number-weighted mean volume of lamellar bodies, which is indicative of an increased level of surfactant phospholipid formation. The improved preservation of the surfactant system by Celsior was accompanied by an anti-inflammatory effect, which was reflected by normal levels of polymorphonuclear leukocytes and alveolar macrophages. Improved lung function achieved by Celsior, as compared with EuroCollins, resulted from both enhanced preservation of the intracellular surfactant system and an anti-inflammatory effect.

In this study, we showed that the total amount of intracellular surfactant, determined by a novel unbiased stereological approach [10,11], remained unchanged in lungs preserved with Celsior when compared to control lungs, whereas it was decreased after preservation with EuroCollins. Young and co-workers [19] demonstrated a correlation between biochemical and morphometric parameters in the quantification of intracellular surfactant, i.e. lamellar bodies, so that we can assume that the amount of lamellar bodies corresponded to the biochemical surfactant phospholipid pool in the cells. Since the apical surface fraction of type II cells was unchanged after IR, it can be assumed that exocytosis and endocytosis of surfactant were well balanced. In contrast, the apical cell surface is expected to grow when more surfactant is secreted than recycled, which is based on the finding that the lamellar body membrane is incorporated into the cell surface during exocytosis [20]. Thus, the reduced amount of intracellular surfactant in lungs preserved with EuroCollins, is suggested to reflect a decrease in surfactant synthesis rather than an increase in surfactant secretion. After preservation with Celsior, the size reduction of lamellar bodies was compensated by a greater number, in a way that the total amount of intracellular surfactant stayed in the range of native lung values. This suggests that surfactant synthesis by type II pneumocytes was increased in the Celsior group.

Immunolabelling for SP-A was highly specific showing quite intensive labelling of the tubular myelin. Unlike some other studies [15,21,22], no specific labelling of unilamellar vesicles or lamellar body-like forms could be detected, though occasional labelling occurred. Biochemical analysis revealed that SP-A accounts for about 1% of total lamellar body protein [23,24] and about 4 to 8% of total lung SP-A was suggested to be present in lamellar bodies [23,25]. However, in the rat, lamellar bodies are less well preserved during cryosubstitution procedures than e.g. in human lung tissue [15], which may account for the low labelling density of lamellar bodies for SP-A in the present study.

The increased labelling density of tubular myelin for SP-A after IR was paralleled by an increase in the total amount of tubular myelin, which was highest after preservation with Celsior [9]. Based on the increase in both, SP-A labelling density as well as tubular myelin volume, the total amount of intraalveolar SP-A can be inferred to be enhanced after IR in the Celsior group. SP-A levels were found to be unchanged [26] or even reduced [26,27] in the bronchoalveolar lavage fluid (BALF) from canine lungs after IR. These differences were shown to depend on the duration of ischemia. Without any specific lung preservation, endogenous SP-A as well as intraalveolar surfactant phospholipids dropped significantly in the BALF from rat lungs after 20 hours of cold ischemia and further decreased markedly after 1 hour of reperfusion [28]. Interestingly, the drop in endogenous SP-A could be reversed by instillation of SP-A-enriched as well as SP-A-deficient surfactant [28]. Thus, high intraalveolar phospholipid levels, as were quantified in our model [9], could be a trigger to stimulate the release of endogenous SP-A. This may represent an early protective response that compensates in part for the IR related surfactant inactivation. This protective potential of the lung appears to vanish with extended time of ischemia [26,27], and in the clinical transplant situation [5,7,8] where the declining release of surfactant phospholipids and SP-A may result from yet suboptimal preservation procedures.

Notably, Celsior preserved lungs had almost normal amounts of polymorphonuclear leukocytes and alveolar macrophages, whereas both cell populations were significantly increased in lungs preserved with EuroCollins. Both cell types are well known to release reactive oxygen species (ROS) [29]. ROS, which are formed during early reperfusion, are suggested to inactivate surfactant phospholipids [30]. Additionally, nitration of SP-A was shown to affect its ability to aggregate lipids [31], and oxygen exposure was shown to increase surfactant protein expression [32]. High levels of SP-A were shown to counteract the inhibition of surfactant by serum proteins [33], and to restore the activity of oxidized surfactant *in vitro *[34]. The high protective potential of the Celsior solution has been attributed to the presence of antioxidants such as glutathione and lactobionate, which are thought to counteract the formation of ROS during IR [35]. As multivariate analysis indicates that both low mass of alveolar macrophages and high amount of intracellular surfactant are predictors for lung function parameters, it appears likely that Celsior exerts a dual effect on both infiltrating immune cells and integrity of the surfactant system. Whether the anti-inflammatory effect of Celsior is an indirect result of the improved preservation of the surfactant system, the inactivation of which contributes to an enhanced susceptibility of the lung to inflammation [36], or whether it is a direct effect of its antioxidant components remains to be elucidated.

## Conclusion

In summary, preservation with Celsior increased intraalveolar SP-A levels, stabilized the amount of intracellular surfactant and reduced lung inflammation. In contrast, significant changes of the tubular myelin microstructure and reduction in the amount of intracellular surfactant as well as increased inflammatory cell infiltration occurred in lungs that had been preserved with EuroCollins. We suggest that high intraalveolar levels of surfactant phospholipid and SP-A represent an early protective response directed to compensate for the inactivation of intraalveolar surfactant in the early phase of IR injury. We further suggest that maintenance of alveolar epithelial type II cell function by improved lung preservation will support this inherent protective response during early reperfusion.

## Authors' contributions

AF conceived of and participated in the design of the study, performed the quantitative immunolabelling, supervised the stereological analyses, and drafted the manuscript. ST carried out the stereological and statistical analyses. HF participated in the design of the study, the statistical analysis, and drafted the final version of the manuscript. MO participated in the design of the study and in the drafting of the manuscript. TWi performed the extracorporeal ischemia/reperfusion experiments and participated in the design of the study. TWa participated in the design of the study and supervised the animal experiments. JR participated in the design of the study and supervised the ultrastructural investigations. All authors read and approved the final manuscript.

## Supplementary Material

Additional File 1 Table - Stereological Analysis of Intracellular SP-A labelling. File contains an additional table, which summarizes the results of the determination of the relative labelling index. Click here for file
