# Supplementary material for: Improved lung preservation relates to an increase in tubular myelin-associated surfactant protein A
Source: Respir Res. 2005 Jun 21;6(1):60. doi: 10.1186/1465-9921-6-60 (PMC1187923; doi:10.1186/1465-9921-6-60)
Supplement: Additional File 1 — Table - Stereological Analysis of Intracellular SP-A labelling. File contains an additional table, which summarizes the results of the determination of the relative labelling index. [file 1465-9921-6-60-S1.doc]

## Table - Stereological Analysis of Intracellular SP-A labeling

| **Group** | **Compartment** | **N_gold_obs.** | **V_V_(comp/cell)** | **N_gold_exp.** | **RLI** |
| --- | --- | --- | --- | --- | --- |
| Control | Cytoplasm/vesicles | 1076±96 | 52.0±1.5% | 712±86 | 1.53±0.07 |
|  | Lamellar bodies | 93±16 | 16.0±2.5% | 210±10 | 0.47±0.05 |
|  | Nucleus | 180±45 | 27.0±2.5% | 375±72 | 0.45±0.09 |
|  | Mitochondria | 19±6 | 4.7±1.2% | 64±20 | 0.30±0.04 |
| EuroCollins | Cytoplasm/vesicles | 1089±10 | 52.7±1.5% | 681±41 | 1.61±0.08 |
|  | Lamellar bodies | 62±20 | 9.3±0.3% | 120±5 | 0.29±0.08 |
|  | Nucleus | 129±44 | 32.7±0.9% | 423±28 | 0.51±0.15 |
|  | Mitochondria | 3±2 | 5.3±0.7% | 70±11 | 0.18±0.03 |
| Celsior | Cytoplasm/vesicles | 1270±273 | 54.5±1.5% | 848±239 | 1.53±0.11 |
|  | Lamellar bodies | 135±70 | 10.5±3.5% | 176±96 | 0.26±0.08 |
|  | Nucleus | 122±53 | 30.0±4.0% | 448±57 | 0.78±0.03 |
|  | Mitochondria | 0.7±0.5 | 5.5±0.5% | 83±14 | 0.24±0.03 |

*Definition of abbreviations:* N_gold_obs.: Observed intracellular distribution of gold particles labeling for SP-A over 172 type II pneumocyte profiles in 9 rat lungs. V_V_(comp/cell): Relative volume fractions of the cell compartments. N_gold_exp.: Expected distribution of gold particles, which would occur if the gold particles were scattered randomly over the cells, according to the relative volume of the cell compartments. RLI: relative labeling index, which is the observed value divided by the expected value. An RLI of 1 indicates random labeling, while values higher than 1 indicate nonrandom (preferential or specific) labeling. Statistical testing by χ^2^-analysis reveals a highly significant nonrandom labeling for every group (χ^2^: p<0.001). Means ± sem; differences between groups are not significant (one-way ANOVA, p<0.05)

**Method for calculation of RLI**

The observed distribution of gold particles was compared with an expected distribution, which would occur if the gold particles were scattered randomly over the cells, according to the relative volume of the cellular compartments V_V_(comp/cell) the gold particles were associated with. The expected number of gold particles for each compartment (N_gold_expected) was determined according to the formula: N_gold_expected = N_gold_observed x V_V_(comp/cell). The relative labelling index (RLI) was determined for each compartment as the observed value divided by the expected value: RLI = N_gold_observed / N_gold_expected. An RLI of 1 would therefore indicate random labelling, while values higher than 1 would indicate non-random (preferential or specific) labelling.
